# Silencing of miR169a improves drought stress by enhancing vascular architecture, ROS scavenging, and photosynthesis of *Solanum tuberosum* L

**DOI:** 10.3389/fpls.2025.1553135

**Published:** 2025-03-20

**Authors:** Ziqian Lei, Xingyuan Zhang, Ming Wang, Jun Mao, Xinxi Hu, Yuan Lin, Xingyao Xiong, Yuzhi Qin

**Affiliations:** ^1^ College of Horticulture, Hunan Agricultural University, Changsha, Hunan, China; ^2^ Engineering Research Center for Horticultural Crop Germplasm Creation and New Variety Breeding, Ministry of Education, Changsha, China; ^3^ Yuelushan Laboratory, Changsha, Hunan, China

**Keywords:** potato, miR169a, vascular, drought stress, NF-Y

## Abstract

Vascular bundles regulate water balance, nutrient uptake and transport, and stress responses, ultimately influencing the yield and quality of crops. However, our understanding of the genetic functions of microRNAs (miRNAs) during vascular development remains limited. In this research, the role of miR169a in potatoes was studied. Silencing StmiR169a in potatoes promoted vascular bundle formation, resulting in not only upright and robust stems but also longer roots and more extensive root systems. Histological analysis revealed a significant increase in the number of xylem vessels in the vascular bundles of stems and roots of RNAi-mediated miR169a lines (STTM169). Silencing miR169a led to higher water use efficiency, enhanced photosynthesis rates, elevated enzymatic antioxidant activity, and reduced levels of reactive oxygen species (ROS), thereby enhancing the drought resistance of potatoes. However, overexpression of miR169a lines (OE169a) showed the opposite effects. The nuclear factor Y subunit NF-YA3 was identified as a target gene of StmiR169a. The miR169a/NF-YA3 module may be involved in the regulation of potato vascular bundle development and the response to drought stress.

## 1 Introduction

Modifications in xylem morphology and structure can significantly affect water transport and disrupt the equilibrium between shoot and root growth. Pieter jan De Bauw (De Bauw et al., 2019) found that drought tolerance was related to the thickness of xylem vessels and root morphology in rice, suggesting that these traits could be useful in drought resistance breeding in rice. Potatoes are considered susceptible to drought due to their shallow root system. Traits such as open stem-type canopies and a robust root system could enhance light penetration and improve water absorption, which are associated with increased photosynthesis and drought tolerance in potatoes (Hill et al., 2021). Changes in xylem vessel density and diameter have been connected with drought tolerance (Aliche et al., 2020). Additionally, the stem lodging increases temperature and moisture, rendering potato plants susceptible to diseases such as late blight and gray mold. Therefore, dwarfed genotype has been prioritized (Khush, 1999). However, a minimum plant height is required for sustained high yield (Berry et al., 2004; Shah et al., 2017). These studies indicate that the morphology and structure of vascular bundles are associated with both biotic and abiotic stresses in crops.

MicroRNAs (miRNAs), such as miR165/166, have been discovered to modulate the expression of genes involved in vascular tissue development (Carlsbecker et al., 2010; Miyashima et al., 2011). MiR397 and miR857 are respectively involved in lignin biosynthesis and secondary growth of vascular tissues in poplar and *Arabidopsis thaliana* (Lu et al., 2013; Wang et al., 2014; Zhao et al., 2015). MiR528 negatively regulates the abundance of ZmLAC3 and ZmLAC5 mRNA, influencing lignin biosynthesis and lodging resistance in maize under N-luxury conditions (Sun et al., 2018). Moreover, different members of miR164, miR167, miR168, miR390, miR159, miR162, miR171, miR472, miR482, miR166, miR169, miR396, and miR1450 exhibit diverse expression patterns at different stages of vascular tissue formation (Kim et al., 2005; Tang et al., 2016; Wang et al., 2022). Hence, miRNAs have emerged as crucial regulators of vascular tissue development and deserve further exploration. AtNF-YA5, highly expressed in vascular tissues and guard cells, is regulated by drought stress both transcriptionally and post-transcriptionally via miR169 (Li et al., 2008). MiR169/NFYA is highly conserved in maize, tomato, *A. thaliana*, and *Brassica napus* (Luan et al., 2015; Zhang et al., 2022; Rao et al., 2020; Ji et al., 2023; Li et al., 2021). Further studies confirmed that miR169 confers plants with oxidative stress tolerance and maintains reactive oxygen species (ROS) homeostasis through an ABA-dependent pathway (Ji et al., 2023). Hence, investigating the function of miR169 in modulating vascular development, water homeostasis, oxidative stress response, and drought tolerance in crops represents a significant challenge.

Here, short tandem target mimics (STTMs) (Tang et al., 2012) and overexpression technologies were applied to investigate the potential regulator of miR169 on the vascular bundle in potatoes. The results showed that StmiR169a was related to vascular tissue development and lignin synthesis, which affected stem and root function. Silencing of StmiR169 resulted in maintaining ROS homeostasis, enhancing photosynthetic efficiency, and increasing potato production under drought stress, which proposed a new molecular breeding strategy to exploit the potential of miR169 in enhancing crop productivity and stress tolerance.

## 2 Materials and methods

### 2.1 Plant growth

The potato variety E Shu 3 (E3) was used for genetic transformation. *In vitro*-propagated potatoes were cultured *in vitro* using the stem-cutting seedlings in MS medium for 14 days to study the root phenotypic profiles. Potted stem cutting was maintained in a culture chamber for 35 days to assess the phenotype and drought tolerance ability. The culture chamber was incubated at a temperature of 22°C, with a day/night cycle of 16/8 hours and a light intensity of 250 µE m^−2^ s^−1^. This experiment was conducted at the Vegetable Platform of the College of Horticulture, Hunan Agricultural University.

### 2.2 Bioinformatics analysis

Sequence alignment was performed using DNAMAN6 (University of Manchester, http://www.mirbase.org/). A phylogenetic tree was constructed using MEGA11 to determine the evolutionary relationship of pre-miR169 family members. The *cis*-regulatory elements in the promoter region (1,500 bp upstream of the start codon) of the StmiR169 genes were analyzed using the PLANTCARE website (http://bioinformatics.psb.ugent.be/webtools/plantcare/html/).

### 2.3 Construction of expression vector and generation of transgenic potato lines

To silence miR169, an STTM structure was designed with the following sequence: 5′-TCGGCAAGTCActaTCCTTGGCTGGTTGTGTTGTTTATGGTCTAATTTAAATATGGTCTAAAGAAGAAGAATTCGGCAAGTCACTATCCTTGGCTG-3′ (Tang et al., 2012). The pCAMBIA1300 vectors containing the CaMV35S promoter linked with the designed STTM structure were synthesized by the laboratory of Academician Zhu Jiankang at the Shanghai Research Center Group of Plant Adversity Biology, Chinese Academy of Sciences.

For overexpression of StmiR169a, a 165-bp fragment of genomic DNA containing the flank sequence of StmiR169a was amplified and inserted into the PBI121 vector, driven by the CaMV35S promoter.

*Agrobacterium tumefaciens*-mediated transformation was performed on potato E3 (WT) (Si et al., 2003). Over 20 independent transgenic events were generated. The StmiR169 silencing transgenic plants and overexpressing StmiR169a transgenic plants were named STTM169 and OE169a, respectively. Primer sequences are listed in the **Supplementary Table**.

### 2.4 MiRNA extraction, qPCR, and 5′RLM-RACE

Small RNA extraction, first-strand cDNA synthesis of mature miR169, and stem-loop RT-qPCR were performed according to Luan et al (Yin et al., 2021). For stem-loop RT-qPCR, the U6 RNA was used as an internal standard. Relative expression values were calculated using the 2^−ΔΔCT^ method.

Total RNA extraction, cDNA synthesis of pre-miR169, and RT-qPCR were performed using kits from Accurate Biotechnology (Guangzhou, China). The potato U6 RNA was selected as an internal reference, and the relative expression levels were determined via the 2^−ΔΔCT^ method.

5′-RNA ligase-mediated rapid amplification of cDNA ends (5′RLM-RACE) was performed using total RNA from WT and OE169a as described (Yu et al., 2020). Primer details are provided in the **Supplementary Table**.

### 2.5 Histological analysis of vascular bundles

Tissue samples of stems, roots, and main leaf veins of 25-day-old plants were fixed in Formalin-Aceto-Alcohol (FAA) fixative in 70% ethanol and dehydrated through a graded ethanol series (70%, 80%, 90%, and 100%). Subsequently, the samples were treated with xylene and embedded in paraffin (Sigma-Aldrich, St. Louis, MO, USA). Cross-sections of the stems and roots were obtained using a paraffin slicer. The paraffin sections were stained with a 0.1% (w/v) saffron solid green kit (Solarbio, Beijing, China) and observed under a microscope (Leica, Wetzlar, Germany). Xylem vessels and phloem cells in vascular bundles of stems, roots, and main leaf veins were measured using ImageJ.

### 2.6 The drought treatment and restoration

The 25-day-old potato seedlings of WT, STTM169, and OE169a were treated with drought for approximately 7 days when wilting was observed. Once wilting was observed, watering was resumed until the plants returned to their normal condition. Photosynthesis measurements were taken before drought stress, on the seventh day of the drought stress, and 2 days after rehydration. Simultaneously, root/stem/leaf samples were collected and stored at −80°C for subsequent lignin, H_2_O_2_, malondialdehyde (MDA) contents, and antioxidant enzyme activity analyses.

### 2.7 Measurements of photosynthetic parameters

The rate of photosynthesis was measured by LI-6400 (LI-COR, Lincoln, NE, USA). Plants were placed in the chamber of the gas exchange system and illuminated with a photosynthetically active radiation (PAR) source. The concentration of CO_2_ in the chamber was adjusted to the desired level (400 ppm). The rate of CO_2_ uptake and O_2_ release by the plant was measured, which provided information on the photosynthesis (*Pn*) rate. Additionally, intrinsic water use efficiency (*WUEi*) and instantaneous water use efficiency (*WUEt*) were also calculated (Zhou et al., 2024).

### 2.8 Estimation of lignin, H_2_O_2_, MDA content, and antioxidant enzyme activities

The lignin content was measured as described by Hu (Si et al., 2003). The contents of leaf hydrogen peroxide (H_2_O_2_) and MDA were determined using the kit of Solarbio Ltd. (Beijing, China). H_2_O_2_ content was quantified as reported by Hao et al (Yin et al., 2021), and MDA content was determined similarly to Xia (Hu et al., 2023). Catalase (CAT) enzyme activity was assayed as previously stated (Shin et al., 2018). Total superoxide dismutase (SOD) activity was determined as reported previously (Hao et al., 2021), and peroxidase (POD) activity was determined using the kit of Solarbio Ltd.

### 2.9 Measurement of yield under water-limited conditions

Nine treatments were performed in this test, which were randomly arranged in cultivation tanks separated from each other. The cultivation area of each treatment was 5 m^2^. Potato seedlings were planted approximately 20 cm apart with rows 25 cm apart. The initiation of the water treatment occurred 20 days post-sowing. The treatment conditions comprised CK (with a soil volumetric water content, θw, ranging from 65% to 75%), moderate drought (θw, 40%–50%), and severe drought (θw, 20%–30%). A soil moisture meter (PMS710, from China) was utilized to monitor the soil volumetric water content (θw). In each pot, three measurement points were selected. A 12-cm probe was chosen for θw monitoring, and if necessary, daily rehydration was carried out. Plot yield was compared at maturity.

### 2.10 RNA sequencing and differentially expressed gene analysis

The total RNA of stems and roots was extracted from OE169a, STTM169, and WT of 25-day-old seedlings. Sequencing methods, Fragments Per Kilobase Million (FPKM) calculation, and Gene Ontology (GO) and Kyoto Encyclopedia of Genes and Genomes (KEGG) analyses were performed according to previous protocols (Kanehisa et al., 2007; Roberts et al., 2011; Love et al., 2014; Kim et al., 2015; Anders et al., 2015; Chen et al., 2018; Consortium, T.G.O, 2019). RNA-seq data have been deposited in the National Center for Biotechnology Information (NCBI) BioProject database under accession number (PRJNA1029510).

### 2.11 Statistical analysis

Statistical analysis was performed using Microsoft Excel 2016 and SPSS 27.0. All data were expressed as mean standard deviation (± SD) and shown with error bars. One-way ANOVA was used for significant differences between the means of the two groups (* p ≤ 0.05, ** p ≤ 0.01, and ***p ≤ 0.001). Duncan’s method was used for purposes of significance analysis for multiple comparisons (p ≤ 0.05). Charts were drawn using OriginPro 8.

## 3 Results

### 3.1 Characterization of isoforms and expression profile of StmiR169s in potato

**Figure 1:**
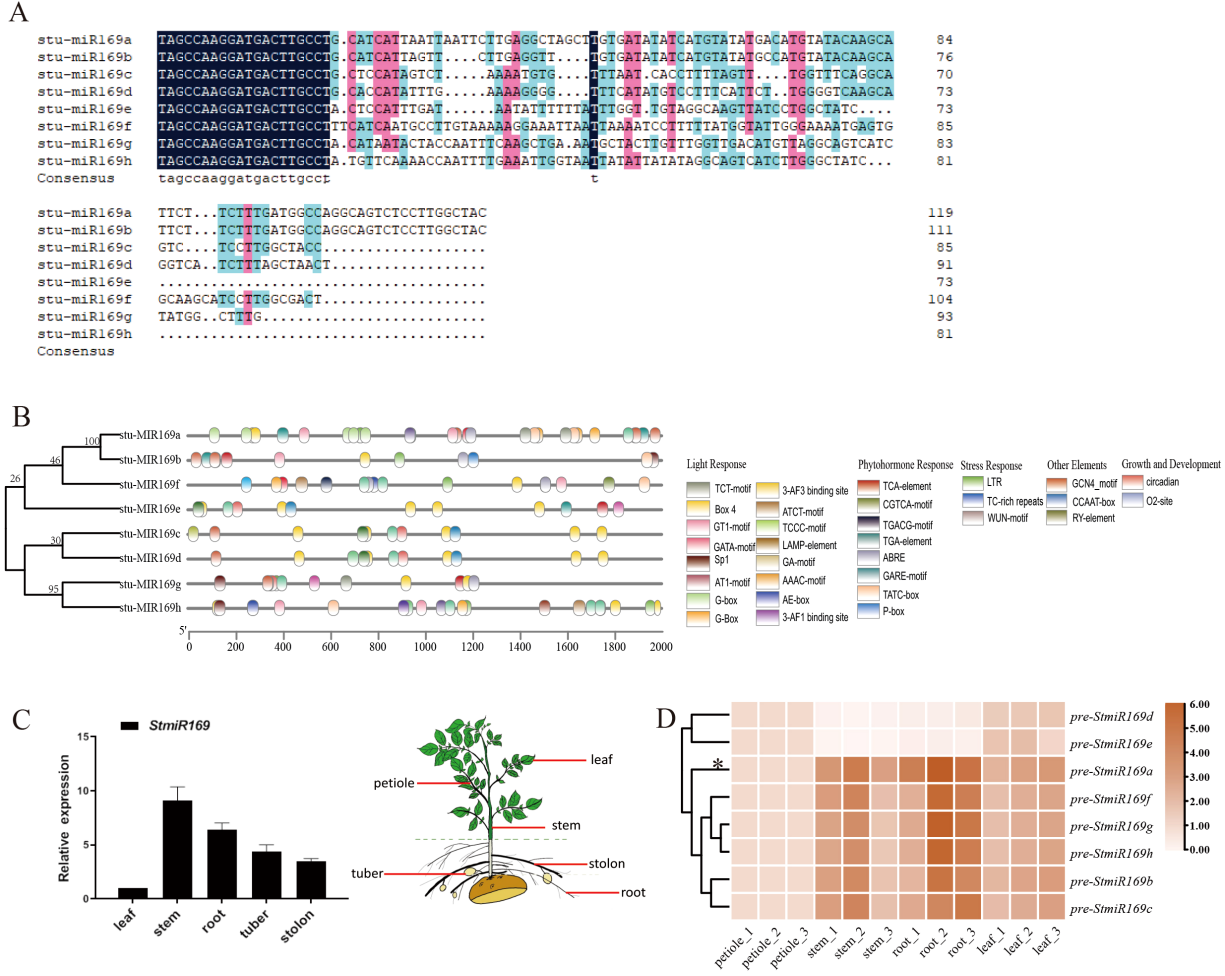
Bioinformatics analysis and expression profile of StmiR169. **(A)** Sequence alignment of potato pre-miR169 and mature miR169 is indicated in blue above the alignments. **(B)** Phylogeny analysis of StmiR169 precursors performed using MEGA11. Diagram showing locations of the *cis*-acting elements in promoters of eight StmiR169s. **(C)** Different expressions of mature *miR169* in leaves, stems, roots, tubers, and stolons by stem-loop RT-qPCR. **(D)** Differential expressions of *pre-miR169a/b/c/d/e/f/g/h* in leaves, petioles, aerial main stems, underground main stems, and roots by RT-qPCR. Heatmap of calculated z-score for each *pre-miR169* plotted by means of a color scale.

To identify potential regulator miR169 in the vascular formation of potatoes, eight potato pre-miR169 genes (a–h) situated on potato chromosomes 3, 7, and 8 were investigated using the miRBase online database (Griffiths-Jones et al., 2008). Sequence alignment revealed high identity in the 5′ sequences of the eight pre-miR169s but significant divergence in length and sequence outside this region. Notably, all eight pre-miR169s produced a single mature miRNA: 5′-TAGCCAAGGATGACTTGCCT-3′ (**Figure 1A**). Phylogenetic analysis grouped the eight pre-miR169s into two major clades (**Figure 1B**). Analysis of *cis*-elements in the StmiR169 promoters revealed abundant light-responsive, hormone-responsive, and stress-related elements (e.g., LTR, MYC-like, MYB, and W-box) (**Figure 1B**).

To evaluate tissue-specific regulation mediated by *StmiR169*s, *StmiR169* expression in different tissues was investigated, including leaves, stems, stolons, tubers, and roots, by RT-qPCR. The results show that mature *miR169* and *pre-miR169* transcripts were present in all tissues; however, expression levels in the stem and root were higher compared with those in other tissues (**Figures 1C, D**). Compared with the expression of other *pre-miR169*s, the *miR169a* was the highest in potato vascular tissue.

### 3.2 Silencing of StmiR169a enhances lodging resistance by promoting rooting and vascular bundle formation in potato

**Figure 2:**
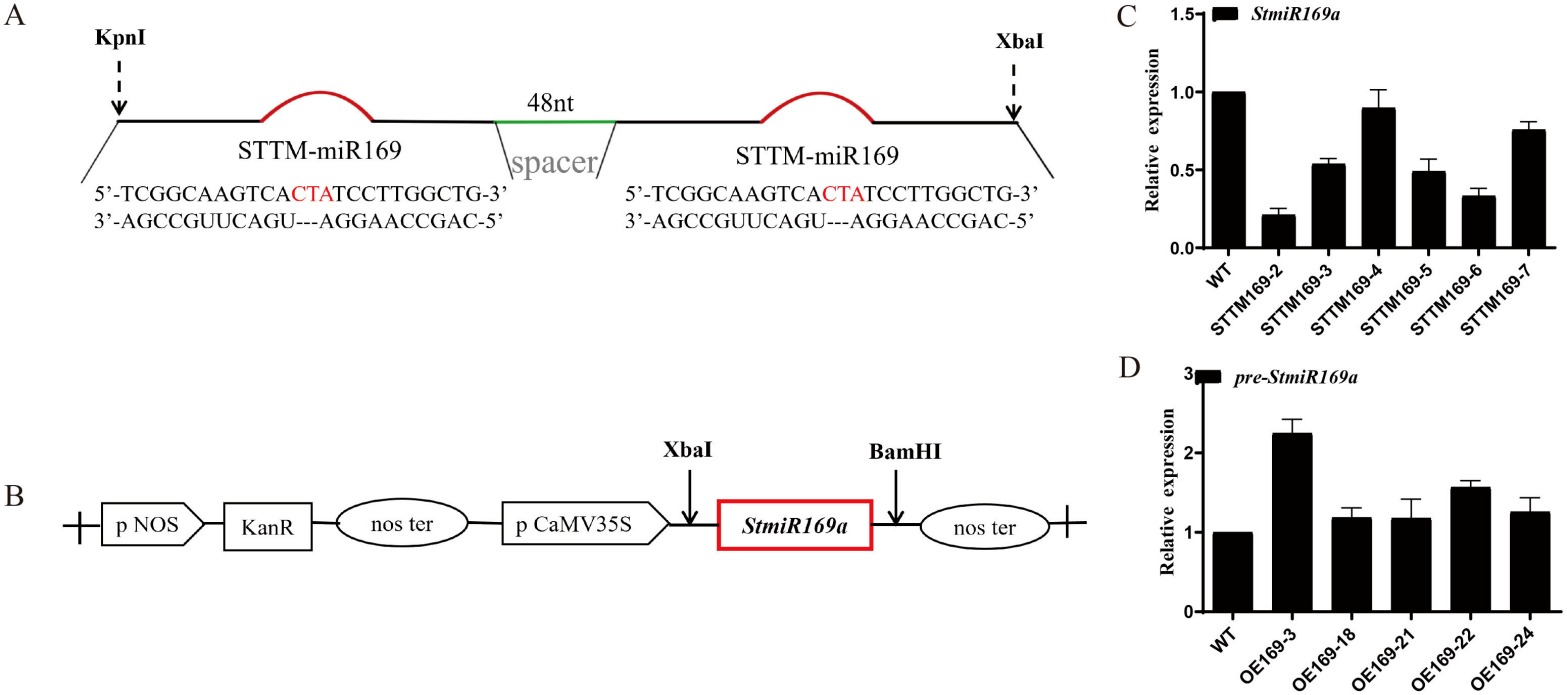
Construction of plant expression vector and potato transformation. **(A)** The short tandem target mimic (STTM) construct was used for silencing *stu-miR169-5p*. **(B)** A 165-bp DNA fragment harboring the *StmiR169a* hairpin structure was cloned into the pBI121 binary vector under the CaMV 35S promoter. Expression levels of miR169-5p in WT and transgenic lines STTM169 **(C)** and OE169a **(D)**.

**Figure 3:**
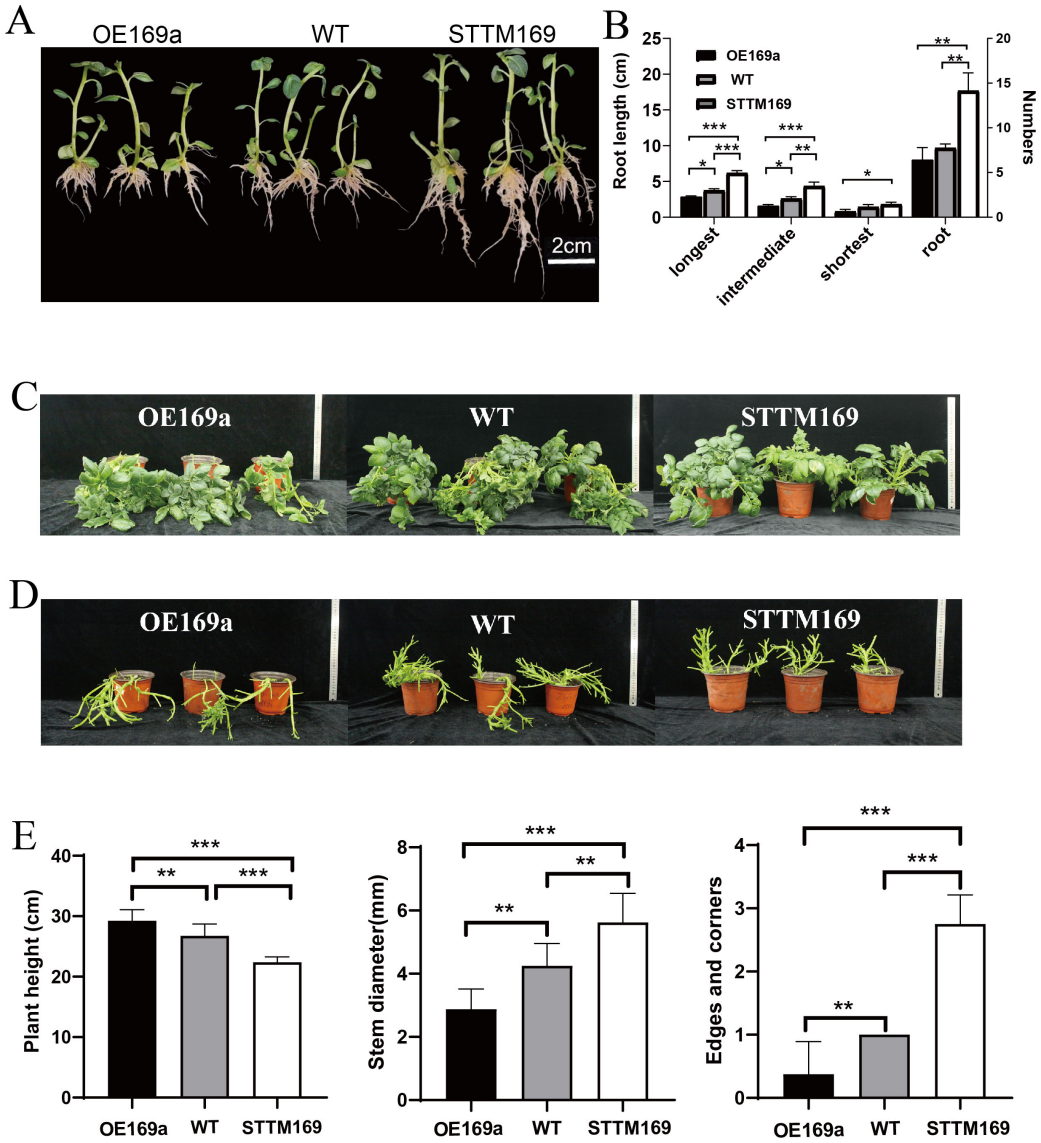
Phenotype of WT, STTM169, and OE169a. **(A)** Seedlings in MS medium for 14 days. **(B)** The root characteristics of 20 plants of each material. **(C)** The 35-day-old WT and STTM169, OE169a foliage structure, and two to three transgenic plants were selected for each independent transgenic line. **(D)** Growth habits in pot. **(E)** Quantification of plant height, stem diameter, and the number of edges and corners in 35-day-old potted plants. Significant differences between the means of the two groups were evaluated by the one-way ANOVA (*p ≤ 0.05, **p ≤ 0.01, and ***p ≤ 0.001).

Six lines of *StmiR169* short tandem target mimic potato (STTM169) and five overexpression *StmiR169a* lines (OE169a) driven by the CaMV35S promoter were obtained (**Figures 2A–D**), and their phenotypes were analyzed. The results showed that compared to WT and OE169a, the STTM169 lines had more and longer roots (**Figures 3A, B**). The OE169a lines were higher in plant height and thinner in the stem, resulting in more severe lodging than STTM169 lines and WT (**Figures 3C–E**).

**Figure 4:**
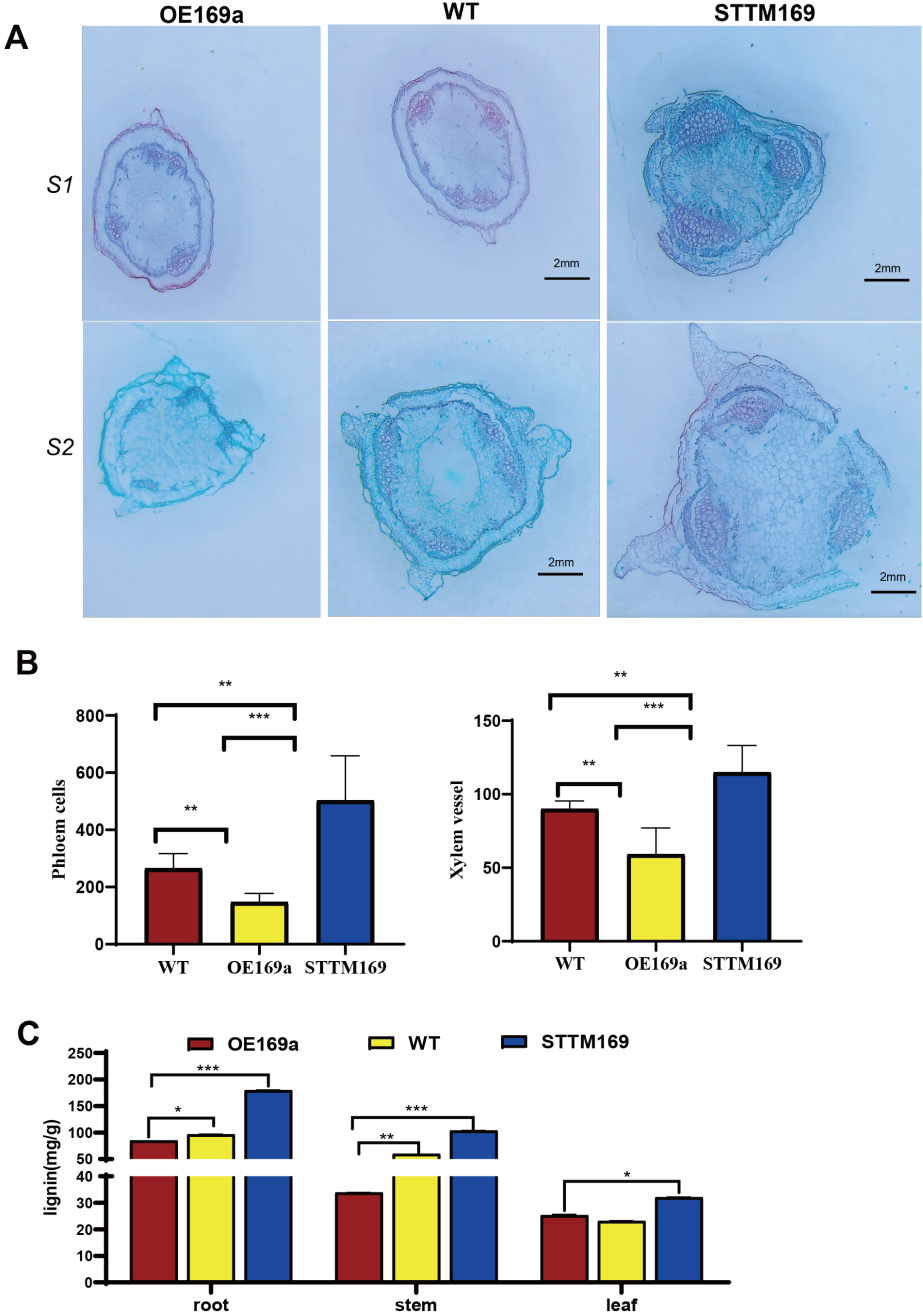
Secondary xylem proliferation and vessel expansion of *StmiR169* isoform mutants. **(A)** Histological observations of stems at the base of plants: S1, the first stem; S2, the second stem. **(B)** Quantification of xylem vessels, phloem cells, and **(C)** lignin content in the base stems of 35-day-old seedlings. Scale bars, 2 mm.

The histological analysis revealed that the area and number of vascular bundles were significantly decreased in the main stems of OE169a compared to STTM169 and WT, and STTM169 exhibited larger and more xylem vessels (**Figures 4A, B**). Additionally, similar phenotypes were observed in roots and leaf venation. The lignin content tests showed that the STTM169 had higher lignin content than WT, while OE169a had the lowest (**Figure 4C**).

### 3.3 StNFYA3 is a target gene of StmiR169a

**Figure 5:**
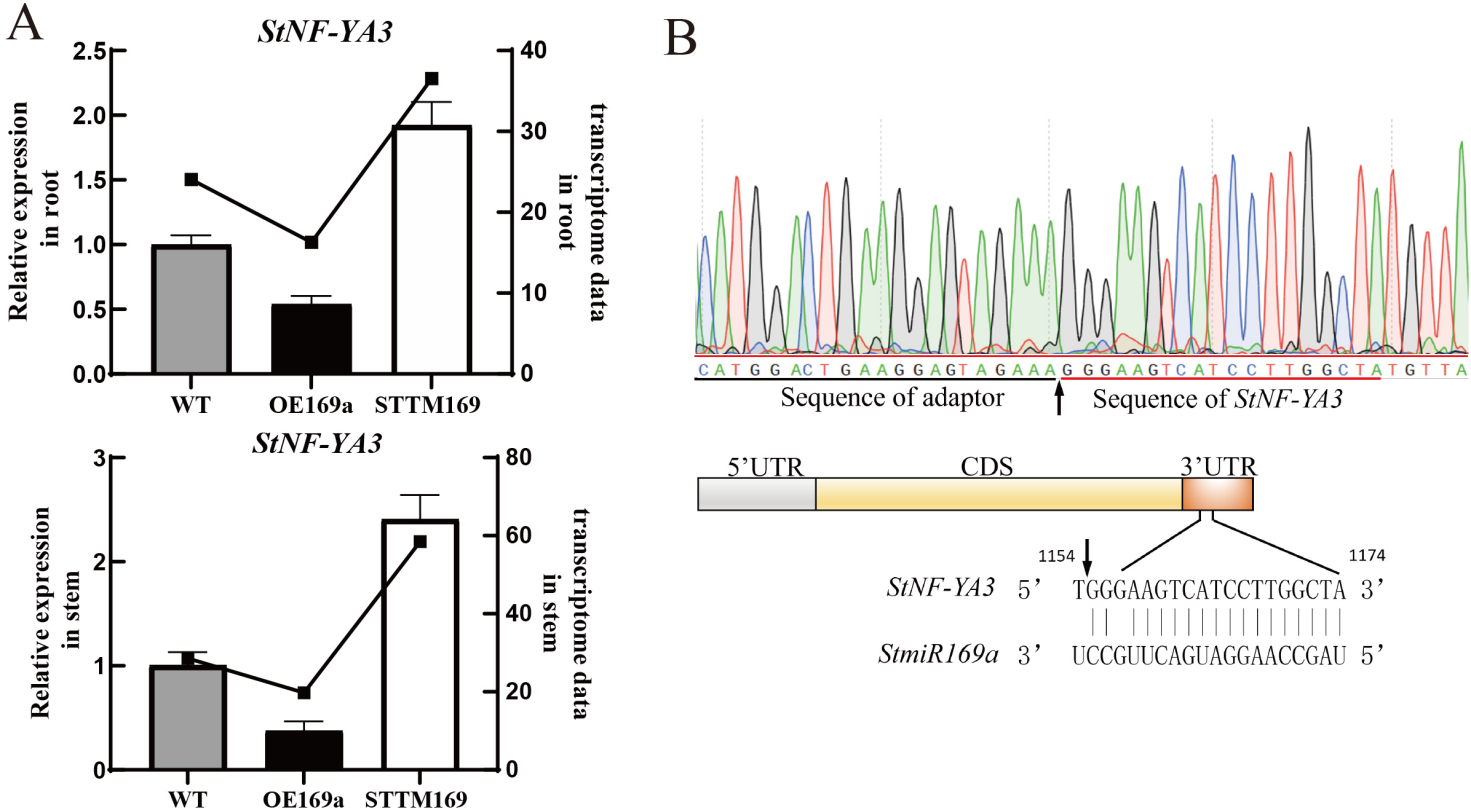
Cleavage site identification of *StNF-YA3* mRNA. **(A)** RT-qPCR analysis of *StNF-YA3* genes in the WT, STTM169, and OE169a. **(B)** The arrow indicates the cleavage site identified in OE169a histocultures by 5′-RLM-RACE.

To further explore the regulatory mechanism of *StmiR169*, the transcriptome of stems and roots from STTM169, OE169a, and WT were compared (PRJNA1029510). In the stem and root, the transcription factor StNF-YA3, which is suspected to be the target gene of StmiR169a, was significantly upregulated in STTM169 and significantly decreased in OE169a, respectively (**Figure 5A**). The 5′-RLM-RACE assay was performed to identify the StmiR169a-directed cleavage site. Twenty clones of 5′-RLM-RACE were sequenced, and the first sequencing reads of 20 clones were at 1,155 bp of StNF-YA3 mRNA and located at 2 bp in the miR169a/StNF-YA3 mRNA complementary site (**Figure 5B**).

### 3.4 Silencing StmiR169a increases antioxidant activities in potatoes under drought stress

**Figure 6:**
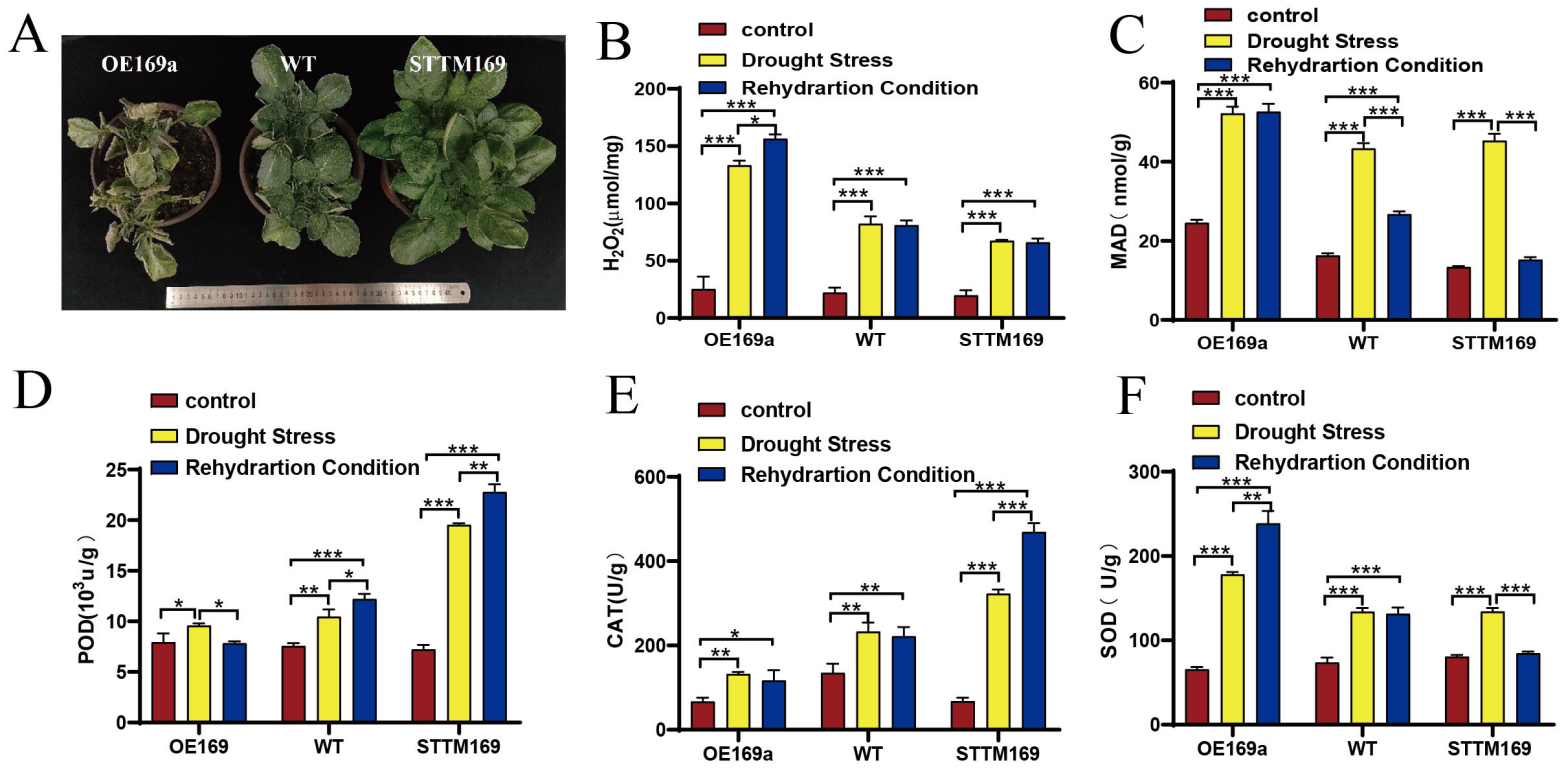
*StmiR169a* related to drought tolerance characteristics. **(A)** Phenotypes of WT, STTM169, and OE169a under drought stress. **(B)** H_2_O_2_ and **(C)** MDA contents and activities of **(D)** POD, **(E)** CAT, and **(F)** SOD were assessed in leaves of WT, STTM169, and OE169a. Significant differences between the means of the two groups were evaluated using the one-way ANOVA (*p ≤ 0.05, **p ≤ 0.01, and ***p ≤ 0.001). MDA, malondialdehyde; POD, peroxidase; CAT, catalase; SOD, superoxide dismutase.

The functional deficiency of *StmiR169* isoforms endowed the STTM169 lines with an inherent ability for higher drought tolerance (**Figure 6A**). The contents of H_2_O_2_ and MDA of STTM169, WT, and OE169a were analyzed, and the findings indicated that the H_2_O_2_ levels of STTM169 were markedly lower than those in OE169a under drought stress (**Figure 6B**). After rehydration treatments, MDA levels in STTM169 declined rapidly; however, OE169a remained at a high level (**Figure 6C**).

After drought and rehydration treatments, the POD activity in STTM169 significantly increased compared to that in WT, while no significant change was observed in OE169a (**Figure 5D**). The activity of CAT was the lowest in OE169a lines, followed by WT, and the CAT activity of STTM169 lines was relatively low without drought and rehydration treatments but significantly increased after drought and rehydration (**Figure 6E**). The SOD activity in WT, OE169a, and STTM169 increased significantly after the drought, but only STTM169 quickly recovered to the level before drought treatment after rehydration (**Figure 6F**).

### 3.5 StmiR169a silencing alleviates the reduction in photosynthesis induced by drought stress

**Figure 7:**
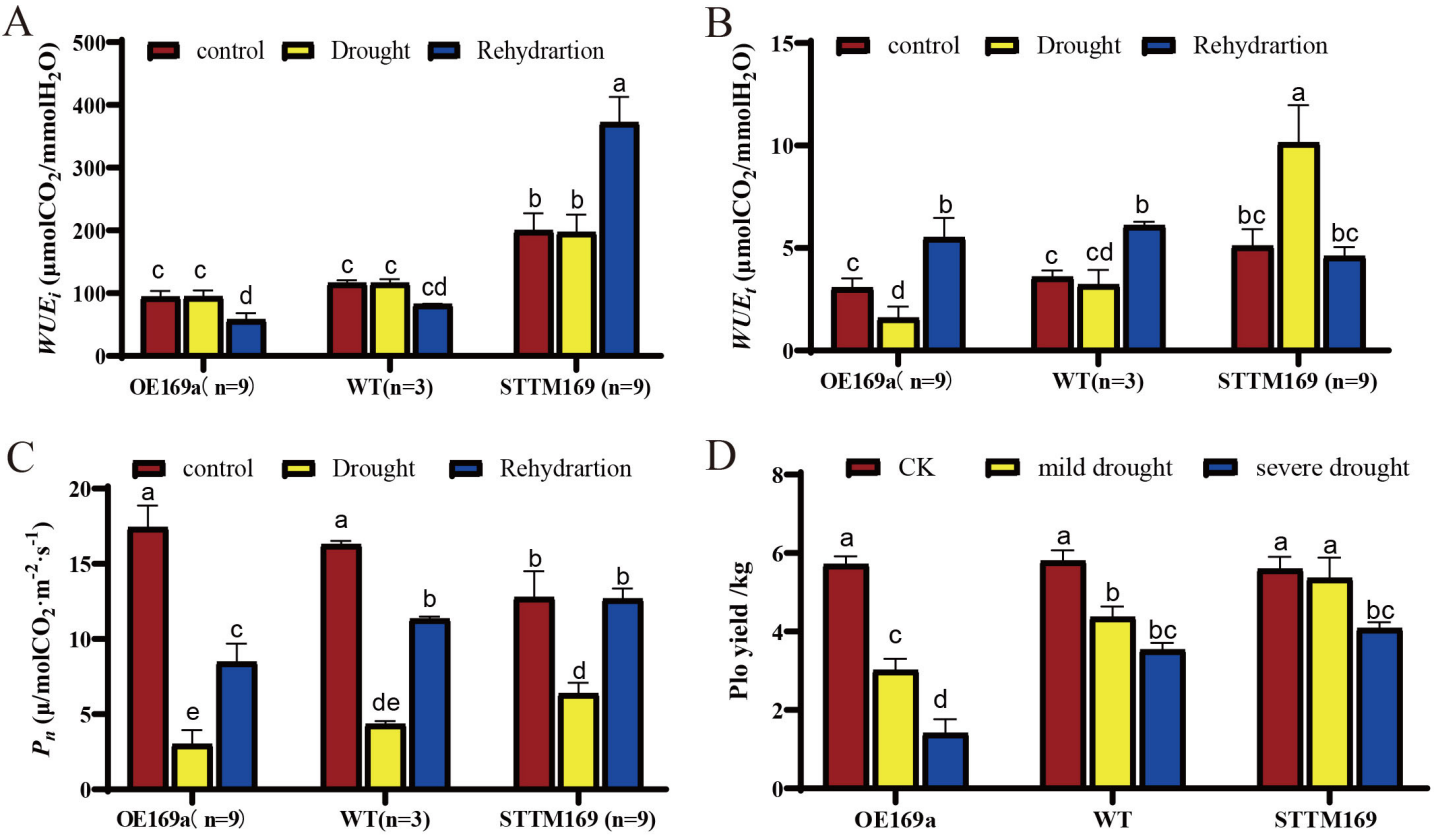
Effects of drought stress on photosynthetic performance. **(A)** The net photosynthetic rate (*Pn*), **(B)** instantaneous water use efficiency (*WUEt*), and **(C)** intrinsic water use efficiency (*WUEi*). **(D)** Yield of potatoes under water-limited conditions. Different letters above columns denote significant differences. The data are mean of three replications. Duncan’s method was used for significance analysis for multiple comparisons (p ≤ 0.05).

Water use efficiency (*WUE*) is the key indicator for measuring the efficiency of plants in utilizing water. STTM169 showed significantly higher *WUEt* and *WUEi* compared to those in OE169a and WT (**Figures 7A, B**). The *WUEt* of STTM169 increased significantly after drought treatment, while that of OE169a and WT decreased. However, the *WUEi* of STTM169 increased quickly and significantly after rehydration, whereas the recovery in OE169a and WT was slower.

The net photosynthetic rate (*Pn*) of the STTM169 (12.78 μmol/m^2^·s) was lower than that of OE169a (17.42 μmol/m^2^·s) and WT (16.29 μmol/m^2^·s) before drought treatment; however, the *Pn* of STTM169 decreased the least after drought treatment. Moreover, the *Pn* of STTM169 recovered to the pre-drought level after 2 days of rehydration, whereas the recovery of *Pn* in OE169a and WT was relatively slow (**Figure 7C**).

The yields of WT, STTM169, and OE169a were measured under normal irrigation, moderate drought, and severe drought. The results showed that under moderate and severe drought conditions, the yields of STTM169 were higher than those of WT and especially higher than those of OE169a plants (**Figure 7D**).

## 4 Discussion

### 4.1 The StmiR169a regulates the formation of vascular system, enhancing lodging and drought resistance in potato

*MiRNA169* is a widespread and highly conserved microRNA across plant species, which regulates the conserved transcription factor NF-YA, and has a role in multiple organ development and biotic/abiotic stress (Zhang et al., 2022; Chen et al., 2024). However, experimental studies on the functions and mechanisms of *miR169*s in potatoes are lacking. There are eight *miR169* family members in potatoes, whose precursors exhibited variations in size and sequence. Unlike *miR169* family members of other species, such as *A. thaliana* and rice, the eight *pre-miR169*s in potatoes are processed to produce only one mature miRNA (**Figure 1A**). The expression level of mature *StmiR169* was higher in stem and root than in other organs, especially *StmiR169a* (**Figures 1C, D**). Intriguingly, the root and stem development of transgenic lines overexpressing *miR169a* was adversely affected and manifested a lodging phenotype, whereas the opposite was the case for transgenic lines with partially disrupted *miR169* (**Figure 3**). The histological analysis revealed that the area and number of vascular bundles were significantly increased in the main stems of STTM169 compared to OE169a and WT. Moreover, STTM169 had more and larger xylem vessels (**Figures 4A, B**). It is evident that the primary cause of the impaired development of the potato vascular system is the overexpression of *miR169*, which is directly associated with the potato’s resistance to lodging.

The well-developed vascular system in STTM169 also promotes the absorption and transport of water in STTM169, thereby improving the water use efficiency under drought stress (**Figures 7A, B**). The higher antioxidant capacity of STTM169 allows for an efficient balance of ROS under drought stress to protect potatoes (**Figure 6**). These findings provided the structural and physiological basis for STTM169’s resistance to drought stress, which eventually improves the STTM169 photosynthetic efficiency and yield under drought stress (**Figures 7C, D**).

The formation of the vascular system in plants is regulated by a complex network. Phytohormones, such as auxins, cytokinins, and gibberellins, are known to be important in vascular formation by regulating cell division, differentiation, and elongation (Lucas et al., 2013; Ohashi-Ito and Fukuda, 2014). The interplay between auxin and cytokinin is crucial for vascular formation, which involves a proper arrangement of the xylem, phloem, and procambium (De Rybel et al., 2014; Zhao et al., 2015). The miR169 family plays an important role in hormone-mediated signaling pathways (Guo et al., 2005). The KEGG enrichment analysis stem and root of WT (E3)-STTM169 or WT (E3)-OE169a in this study also indicates an abnormally active hormonal signaling response (sot04075); after the silencing of miR169a, auxin signaling transduction may be enhanced, thereby promoting the development of vascular structures and improving the lodging resistance and water transport efficiency of plants (**Supplementary Figure 1**). Understanding the mechanism of lodging resistance and drought tolerance modulated by *StmiR169a* can help us in improving crop production.

### 4.2 The StmiR169a target NF-YA3 regulated multiple biological processes

The nuclear factor Y (NF-Y) transcription factor is a heterotrimeric complex consisting of three subunits, namely, NF-YA, NF-YB, and NF-YC (Zemzoumi et al., 1999). Each subunit is encoded by a set of genes. The main targets of *miR169*s are genes of NF-YA (Luan et al., 2015; Zhang et al., 2022; Rao et al., 2020; Ji et al., 2022; Li et al., 2021). There have been reports that *miR169* mediates the formation of the vascular system, leaves, and roots by regulating NF-YA (Combier et al., 2006; Luan et al., 2014). Our results of transcriptome sequencing and RT-qPCR analysis showed that compared to the WT, the NF-YA3 gene was significantly upregulated in STTM169 and significantly downregulated in OE169a (**Figure 5A**). The 5′-RLM-RACE confirmed that *StmiR169a* directly cleaves NF-YA3 (**Figure 5B**). Abundant light-responsive and plant hormone-responsive elements were identified within the promoter regions of *StmiR169* genes. Moreover, the significant enrichment of GO terms related to biological process included “defense response” and “jasmonic acid, ethylene, cytokinin and salicylic acid activated signaling pathway”; those of the cellular component included “cell wall”, “plasma membrane”, and “integral component of membrane”; and those of molecular function included “DNA-binding transcription factor activity”, “sequence-specific DNA binding”, “heme binding”, “oxidoreductase activity”, “calmodulin binding”, “iron ion binding”, and “MAP kinase activity” in the STTM169 vs. OE169a, WT vs. STTM169, and WT vs. OE169a groups (**Supplementary Figure 1**). Moreover, the top four KEGG enrichment pathways were “Plant hormone signal transduction”, “MAPK signaling pathway”, “Phenylpropanoid biosynthesis”, and “ABC transporters” (**Supplementary Figure 2**). Furthermore, the top four KEGG pathways were “Environmental adaptation”, “Carbohydrate metabolism”, “Signal transduction”, and “Biosynthesis of secondary metabolites” (**Supplementary Figure 3**). Collectively, transcriptome sequencing results suggest that StmiR169/NF-YA3 is involved in a variety of biological processes, which may include enhancing antioxidant defenses, regulating hormone signaling, improving water absorption and transport capacity, and increasing the efficiency of photosynthesis. However, the regulatory mechanisms underlying these processes require further investigation.

This study disclosed that STTM169 in potatoes not only augmented drought resistance but also enhanced the plants’ capability to scavenge ROS in comparison with WT plants and OE169a. This augmented resistance was accompanied by an improved vascular bundle and root system development, higher water use efficiency, and elevated levels of photosynthesis in the transgenic plants STTM169 under drought stress. Additionally, it was discovered that *StmiR169a* targeted the *NF-YA3* gene, thereby transcriptionally repressing its expression. On the whole, the *StmiR169a*/*NF-YA3* module assumes a pivotal role in enhancing drought tolerance in potatoes by regulating the development of potato vascular bundles. These results validate the functional significance of the *StmiR169* gene and propose it as a promising candidate for the development of novel drought-resistant potato varieties through genetic engineering and response to drought stress.

## Data Availability

The datasets presented in this study can be found in online repositories. The names of the repository/repositories and accession number(s) can be found below: https://www.ncbi.nlm.nih.gov/genbank/, PRJNA1029510/.
