# Supplementary material for: Silencing of miR169a improves drought stress by enhancing vascular architecture, ROS scavenging, and photosynthesis of Solanum tuberosum L
Source: Front Plant Sci. 2025 Mar 20;16:1553135. doi: 10.3389/fpls.2025.1553135 (PMC11965352; doi:10.3389/fpls.2025.1553135)
Supplement: Supplementary file 1 [file DataSheet1.docx]

Supplementary Material

## Primers

The nucleotide sequences of all primers used in this study are listed in Supplementary Table.

**Supplementary Table**

| Primer name | 5’-3’ | application |
| --- | --- | --- |
| *StmiR169a(+)* | cagtGGATCCTAGCCAAGGATGACTTGCCTGCATCATTAATTAATTCTTGAGGCTAGCT | Synthetic *miR169a* sequence |
| *miR169 (M)* | TAATTCTTGAGGCTAGCTTGTGATATATCATGTATATGACATGTATACAAGCATTCTTC |  |
| *StmiR169a(-)* | cagtGAGCTCAGTAGCCAAGGAGACTGCCTGGCCATCAAAGAAGAATGCTTGTATACAT |  |
| HS-35seq | TTCATTTGGAGAGAACACGGGGGAC | PBI121-*StmiR169a*  constructed expression vectors Identification |
| Pbw2- | GCGATTAAGTTGGGTAACGCCAGGG |  |
| aF | GCTTTCAGTTCAAGAACTAAGCTGTGGAAGTTGTTGTTGTTATGGTCTAGTTGTTGTTGTTATG GTCTAA | STTM169 constructed expression vectors Identification |
| aR | TTTAAATTAGACCATAACAACAACAACTAGACCATAACAACAACAACTTCCACAGCTTACTTCTTAACTG |  |
| bF | TTTAAATATGGTCTAAAGAAGAAGAATATGGTCTAAAGAAGAAGAATAAGTTCAAGAACTAAGCTG TGGAAG |  |
| bR | AATTCTTCCACAGCTTAGTTCTTGAACTTATTCTTCTTCTTTAGACCATATTCTTCTTCTTTAGACCATA |  |
| *miR169-F* | TCTTCGTCAACATGGTGGAGCACGA | PBI121-*StmiR169a* constructed expression vectors Identification |
| *miR169-R* | GCGATTAAGTTGGGTAACGCCAGGG |  |
| *Hyg-F* | TACACAGCCATCGGTCCAGA | STTM169 transgenic plants Identification |
| *Hyg-R* | AGGAGGGCGTGGATATGTC |  |
| *miR-U6-F* | CGCACAAATCGAGAAATGGTC | an internal control for miRNA qRT-PCR |
| *Pre-miR169a-F* | AAGGATGACTTGCCTGCATCATTA | RT-qPCR for pre-miR169s |
| *Pre-miR169b-F* | GGATGACTTGCCTGCATCATTAG |  |
| *Pre-miR169c-F* | GACTTGCCTGCTCCATAGTCT |  |
| *Pre-miR169d-F* | CAAGGATGACTTGCCTGCAC |  |
| *Pre-miR169e-F* | AGCCAAGGAGACGCCACC |  |
| *Pre-miR169f-F* | ACTTGCCTTTCATCAATGCCT |  |
| *Pre-miR169g-F* | GCCAAGGATGACTTGCCTACA |  |
| *Pre-miR169h-F* | GCCAAGGATGACTTGCCTATG |  |
| *miR-U6-F* | CGCACAAATCGAGAAATGGTC | miR-U6-F |
| *miR169-F* | TGTAGCCAAGGATGACTTGCCT | stem-loop RT-qPCR for *miR169* |
| *StNF-YA3-F* | AAGCCACGGGAAAAGCAGTA | qPCR for LOC102578385 |
| *StNF-YA3-R* | GGCAGCAACTAGACCGTGAT |  |
| *Actin-F* | ATTCAAGTATGCCTGGGTGCT | loading control |
| *Actin-R* | GTGGTGGAGTCAATAATGAGGAC |  |
| 5’RACE oligo | CGACTGGAGCACGAGGACACTGACATGGACTGAAGGAGTAGAAA | connector primer |
| RLM-RACE 5’Primer | CGACTGGAGCACGAGGACACTGA |  |
| RLM-RACE 5’Nested Primer | GGACACTGACATGGACTGAAGGAGTA |  |
| *a-NRT1* | AACCAGCCACAATAAACT | reverse transcription primer |
| *a-NRT2* | ACTCTTTGGATTGAGGTAT |  |
| *a-R1* | CTCTTTGGATTGAGGTATTTAAAGCAT | specific primer |
| *a-R2* | CAACCATGCCAGGTTATGGAC |  |

## Supplementary Figures


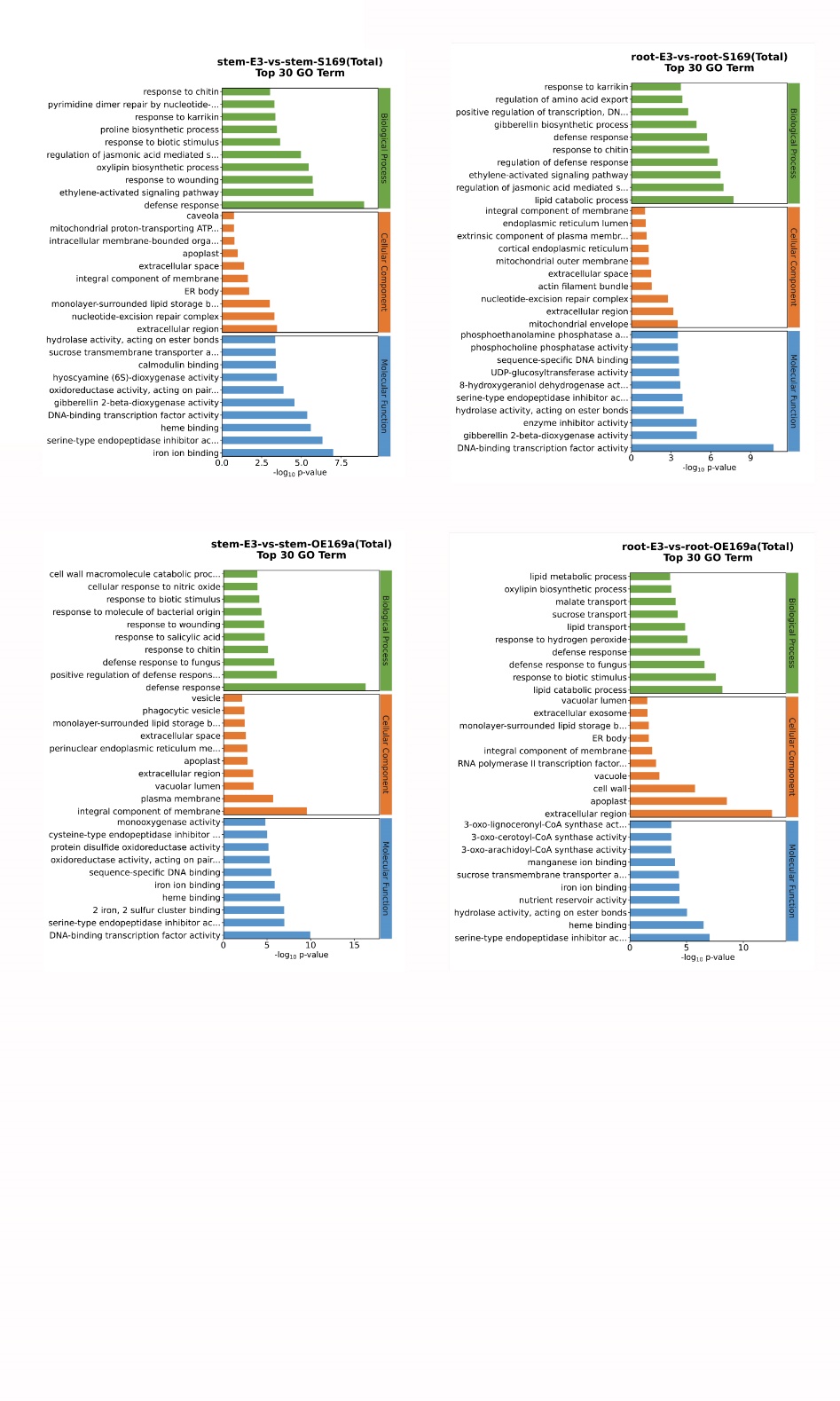


**Supplementary Figure.1. Top 30 GO Term.**

The first row:Stem-E3(WT)-VS-stem-S169(STTM169),root-E3(WT)-VS-stem-S169(STTM169);The second row:Stem-E3(WT)-VS-stem-OE169,root-E3(WT)-VS-stem-OE169.


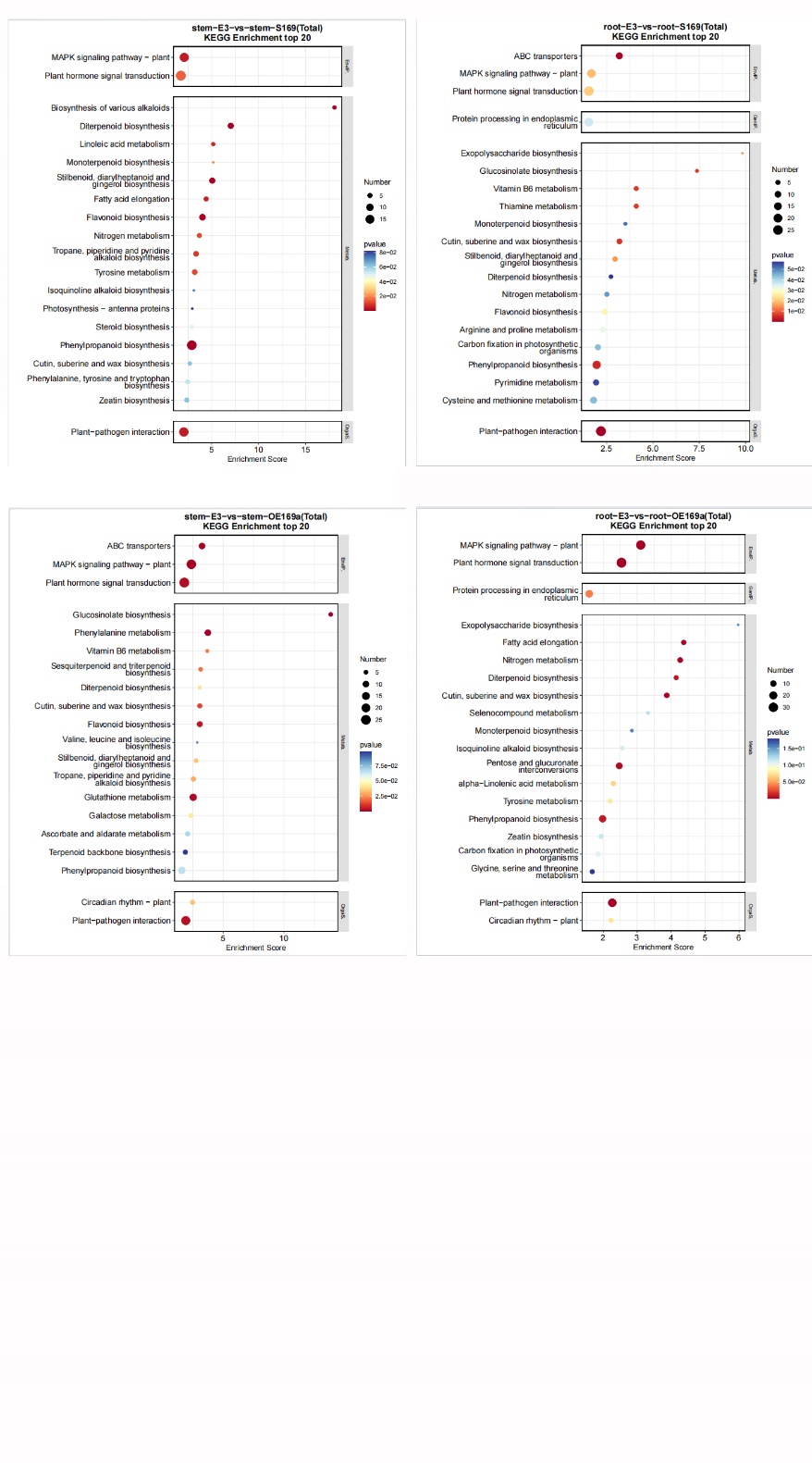


**Supplementary Figure.2. KEGG Enrichment top 20**

The first row:Stem-E3(WT)-VS-stem-S169(STTM169),root-E3(WT)-VS-stem-S169(STTM169);The second row:Stem-E3(WT)-VS-stem-OE169,root-E3(WT)-VS-stem-OE169.


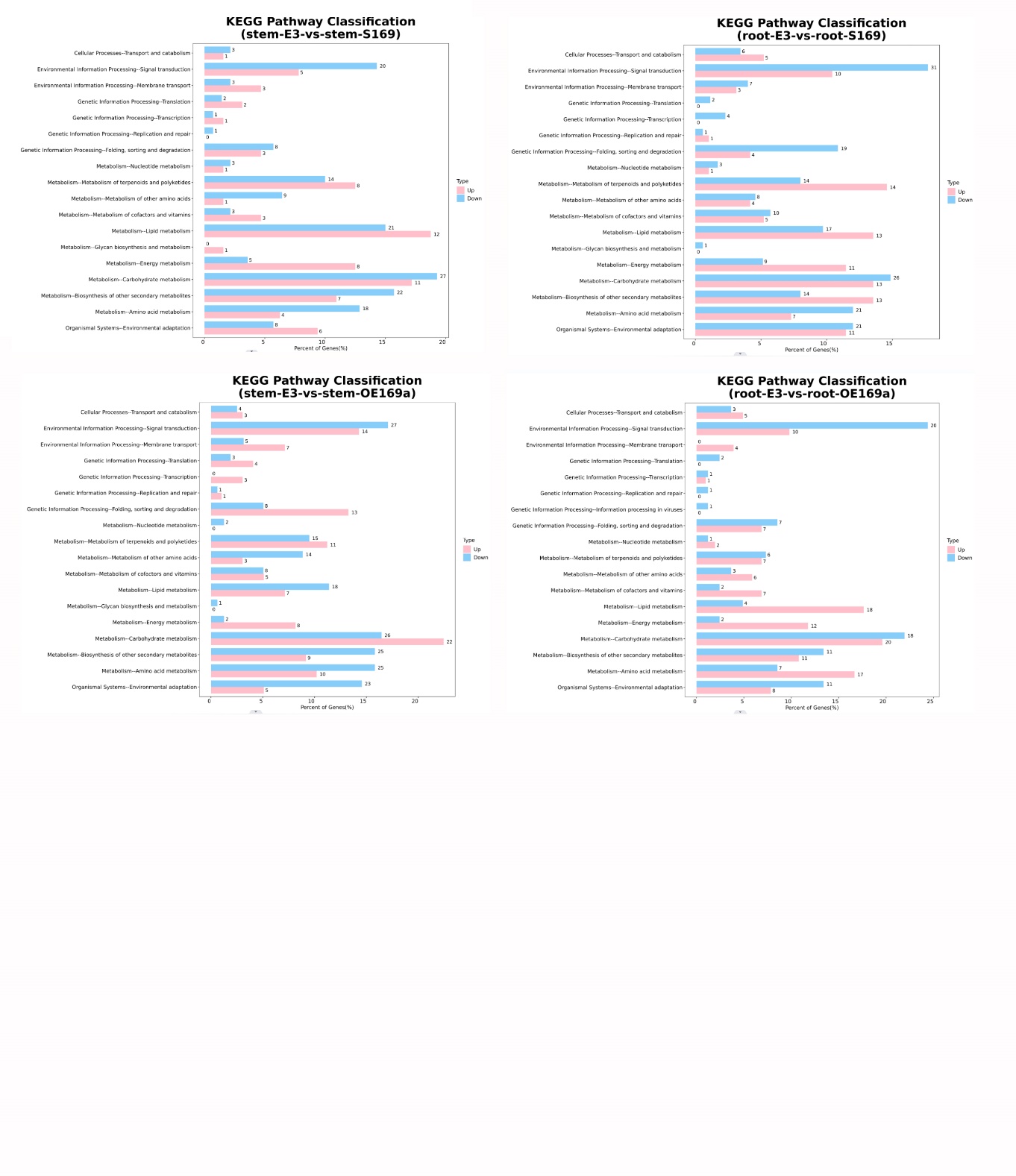


**Supplementary Figure.3. KEGG pathway classification**

The first row:Stem-E3(WT)-VS-stem-S169(STTM169),root-E3(WT)-VS-stem-S169(STTM169);The second row:Stem-E3(WT)-VS-stem-OE169,root-E3(WT)-VS-stem-OE169.
